# Scientometric mapping of the trends, impact, and thematic evolution of scientific production on ehrlichiosis in veterinary medicine

**DOI:** 10.14202/vetworld.2024.2159-2165

**Published:** 2024-09-28

**Authors:** Fran Espinoza-Carhuancho, Julia Medina, Cesar Mauricio-Vilchez, Diego Galarza-Valencia, Roman Mendoza, Josmel Pacheco-Mendoza, Frank Mayta-Tovalino

**Affiliations:** 1 Bibliometrics, Evidence Evaluation and Systematic Review Group (BEERS), Lima, Peru; 2 Research, Innovation and Entrepreneurship Unit, Faculty of Dentistry, Universidad Nacional Federico Villarreal, Lima, Peru; 3 Department of Academic, Faculty of Medical Technology, Universidad Nacional Federico Villarreal, Lima, Peru; 4 Vicerrectorado de Investigación, Universidad San Ignacio de Loyola, Lima, Peru

**Keywords:** bibliometrix, ehrlichiosis, scientometric

## Abstract

**Background and Aim:**

This study focuses on the scientific output of ehrlichiosis, a tick-borne disease that affects a variety of animal species, including dogs, cats, and livestock. Therefore, this study aimed to perform a scientometric mapping of the trends, impact, and thematic evolution of scientific production on ehrlichiosis in veterinary medicine.

**Materials and Methods:**

The study design was descriptive and observational, with a quantitative scientometric approach. This study was based on Scopus data collection and analysis from 2018 to 2023. A literature search was conducted on February 12, 2024, and a total of 200 documents were found, of which 177 were articles, 15 book chapters, and eight reviews. A specific search formula was used to obtain documents. The documents were analyzed using SciVal and Bibliometrix in R Studio, focusing on four key metrics: Scholarly Output, View Count, Field-Weighted Citation Impact, and Citation Count.

**Results:**

This bibliometric study covered the period from 2018 to 2023 and analyzed 200 papers from 84 different sources. The average number of citations was 3595 and the mean age was 3.17 years. A total of 1874 keywords and 1085 authors were identified, with an average of 6.25 co-authors per paper. International co-authorship was present in 23% of the papers. The papers were distributed as articles (177), book chapters (15), and reviews (8).

**Conclusion:**

The combination of these metrics enabled a more complete and accurate assessment of research performance. A total of 1874 keywords and 1085 authors were identified. The thematic evolution from “canine ehrlichiosis” and “Ehrlichia canis” to “dog” and “canine” was observed. Bradford’s and Lotka’s laws were confirmed, with some sources and authors generating most publications.

## Introduction

Ehrlichiosis is a serious infectious disease caused by intracellular, Gram-negative microorganisms of the genus *Ehrlichia*. These bacteria are transmitted by ticks, although they can also be carried by fleas or mites [1]. This disease affects humans and other mammals, and its prevalence may vary depending on the geographic region and the *Ehrlichia* species involved [2]. In dogs, this disease is known as canine *Ehrlichia canis*, specifically caused by the bacterium *E. canis*. This disease is of great importance in veterinary medicine because it is widely distributed worldwide [3]. It is also known by other names, such as canine hemorrhagic fever, canine typhus, and sniffer dog disease [4].

The stages of evolution are determined based on clinical signs and clinicopathological abnormalities observed [5]. Symptoms in naturally infected animals may differ from those observed in experimentally induced infections [6]. Tick-borne diseases are of great public health relevance because multiple outbreaks of new tick-borne diseases and identification of their origins have increased public awareness of these zoonoses [7]. Such is the case of tick-borne disease, Ehrlichiosis, whose causative agents are microorganisms of the genera *Ehrlichia* and *Anaplasma* [8].

Ehrlichiosis follows a course that begins with an incubation period of <10 days, moves to the acute and subclinical phases, and, in certain cases, reaches the chronic phase. This condition affects various animal species, such as dogs, cats, cattle, horses, and some wildlife species [9]. The infection is transmitted through the bite of infected ticks, making it a significant health challenge in various regions of the world, especially in areas where ticks are common. The clinical signs of the disease can vary considerably and include symptoms such as depression, lethargy, weight loss, anorexia, and fever; enlarged lymph nodes and spleen; pale mucous membranes due to anemia, ecchymosis, and black stools [10].

*E. canis* is a bacterium that is globally distributed in tropical regions. The prevalence and recognition of diseases transmitted by ticks to humans and animals have recently increased. This is due to a combination of factors that increase the likelihood of interaction between wild animals, their ectoparasites, domestic animals, and humans. Wild and domestic carnivores are the main carriers of tick-borne zoonotic agents to humans. Notably, pathogens from the *Anaplasmataceae* family (Order *Rickettsiales*) are emerging tick-borne agents with global reach and potential to infect humans [11]. In veterinary medicine, the treatment of Ehrlichiosis usually involves the use of antibiotics such as doxycycline, which are effective in eradicating *Ehrlichia* bacteria from the animal’s body. However, early treatment is essential to avoid serious complications and improve the chances of recovery [12].

Therefore, this study aimed to perform a scientometric mapping of the trends, impact, and thematic evolution of scientific production on ehrlichiosis in veterinary medicine.

## Materials and Methods

### Ethical approval

Given that this research study utilized publicly accessible data, no ethical concerns were raised. This study complied with the guidelines of RAMIBS: Reporting and Measurement of Items for Bibliometric or Scientometric Studies in Health Sciences.

### Study period and location

The study was conducted in February 2024 at the vice-rectorate of research of the Universidad San Ignacio de Loyola, Lima, Peru.

### Literature search

The literature search was conducted on February 12, 2024. A total of 200 papers were found, of which 177 were articles, 15 book chapters, and eight reviews.

### Study design

The study design was descriptive and observational, with a quantitative scientometric approach. This was based on the collection and analysis of data from Scopus from 2018 to 2023. This approach allowed the collection of a large amount of data and quantitative analyses to identify patterns and trends in the field.

### Search formula

The following search formula was used to obtain documents: TITLE-ABS (“Ehrlichiosis” or “canine rickettsiosis” or “human granulocytic ehrlichiosis” or “HGE” or “human monocytic ehrlichiosis” or “HME” or “Ehrlichial infection”) and subjarea (vete) and pubyear >2017 and pubyear <2024.

This formula allowed for the search of documents containing the terms specified in the title or abstract, which are related to the veterinary area (VETE) and which were published from 2018 to 2023.

### Analysis in SciVal and R studio

The documents were analyzed using two main tools: SciVal (https://www.scival.com/home) and Bibliometrix in R Studio. In SciVal, documents were imported, and bibliometric analyses were performed to obtain key metrics, such as the number of citations, author collaboration, and publication trends. The data were then exported to Bibliometrix, an R Studio tool (R version 4.3.2 https://www.r-project.org/) specialized in bibliometric and scientometric analysis. In Bibliometrix, more detailed analyses were performed, including co-citation analysis, keyword co-occurrence analysis, and social network analysis. These analyses identified thematic trends, collaboration patterns, and emerging research areas in the field of study.

### Statistical analysis

The data analysis focused on four key metrics: Views count, field-weighted citation impact (FWCI), and citation count and scholarly output. These metrics provide a comprehensive view of the impact of the literature in the field under investigation. The combination of these metrics allows for a more complete and accurate assessment of research performance.

## Results

This bibliometric study covered the period from 2018 to 2023 and analyzed 200 documents from 84 different sources. The average number of citations was 3595 and the mean age was 3.17 years. A total of 1874 keywords and 1085 authors were identified, with an average of 6.25 co-authors per paper. International co-authorship was present in 23% of the papers. The documents were distributed in articles (177), book chapters (15), and reviews (8) (Table-1).

**Table-1:** Main characteristics.

Description	Results
Timespan	2018–2023
Sources	84
Documents	200
Annual growth rate %	11.84
Average age of documents	3.17
Average citation count per document	3.595
References	8439
Keywords plus	1874
Author’s keywords	578
Authors	1085
Authors of single-authored papers	6
Single-authored docs	6
Co-Authors per document	6.25
International co-authorships %	23
Articles	177
Books	15
Reviews	8

The Universidade Federal de Mato Grosso and the University of California at Davis produced 11 publications each. The Universidade Estadual Paulista Júlio de Mesquita Filho was followed by 10 publications. Despite having only seven publications, the Hebrew University of Jerusalem and North Carolina State University had high field-weighted citation impacts of 3.2 and 3.26, respectively. IDEXX Laboratories was the only corporate entity on the list, with five publications and a FWCI of 2.58 (Table-2).

**Table-2:** Top 10 most representative institutions.

Institution	Country	Scholarly output	Views count	Impact of field-weighted citation	Citation count
Universidade Federal de Mato Grosso	Brazil	11	170	0.7	26
University of California, Davis	US	11	56	2.35	7
Universidade Estadual Paulista Júlio de Mesquita Filho	Brazil	10	172	0.69	29
Hebrew University of Jerusalem,	Israel	7	155	3.2	63
North Carolina State University,	US	7	164	3.26	83
U.P. Pandit Deen Dayal Upadhyaya pashu Chikitsa Vigyan Vishwavidyalaya Evam Go Anusandhan Sansthan	India	7	72	0	0
Universidade Federal Rural, Rio de Janeiro	Brazil	6	65	0.3	12
University of Veterinary and Animal Sciences, Lahore	Pakistan	6	119	1.88	40
IDEXX Laboratories	US	5	109	2.58	60
Aristotle University, Thessaloniki	Greece	4	104	2.33	43

It was found that Greene’s Infectious Diseases of the Dog and Cat, Fifth Edition had 14 publications, followed by Acta Scientiae Veterinariae with 13 publications and Veterinary Practitioner with 11. Despite having only 10 publications, Veterinary World had several views (355) and a FWCI of 1.14. Veterinary Medicine and Science, with only five publications, had the highest field-weighted citation impact (2.67) and the highest number of citations (69) (Table-3).

**Table-3:** Top 10 productive scientific journals.

Source	Scholarly output	Views count	Impact of field-weighted citation	Citation count
Greene’s infectious diseases of the dog and Cat, 5^th^ Edition	14	69	2.37	9
Acta Scientiae Veterinariae	13	154	0.15	9
Veterinary Practitioner	11	125	0.08	4
Veterinary World	10	355	1.14	59
Revista de Investigaciones Veterinarias, Peru	6	224	0.03	1
Ciencia Rural	5	62	0.2	7
Veterinary Clinical Pathology	5	79	0.9	30
Veterinary Medicine and Science	5	114	2.67	69
Veterinary Sciences	5	134	2.21	26
Animals	4	117	1.35	17

The bibliometric study revealed that Jane Emily Sykes of the University of California at Davis led the publication of 10 publications. Mukesh Kumar Srivastava from U.P. Pandit Deen Dayal Upadhyaya Pashu Chikitsa Vigyan Vishwavidyalaya Evam Go Anusandhan Sansthan in India followed with eight publications. Despite having only five publications, Edward Bealmear Breitschwerdt from North Carolina State University had several views (153) and a FWCI of 2.35. Ramaswamy Chandrashekar of IDEXX Laboratories had the highest field-weighted citation impact (2.67) and many citations (45) with only four publications (Table-4).

**Table-4:** Top 10 most productive authors.

Author	Affiliation	Country	Scholarly output	Views count	Impact of field-weighted citation	Citation Count
Sykes, Jane Emily	University of California, Davis	US	10	38	2.58	7
Srivastava, Mukesh Kumar	U.P. Pandit Deen Dayal Upadhyaya pashu Chikitsa Vigyan Vishwavidyalaya Evam Go Anusandhan Sansthan	India	8	82	0.03	1
de Aguiar, Daniel Moura	Universidade Federal de Mato Grosso	Brazil	7	64	0.39	19
Breitschwerdt, Edward Bealmear.	North Carolina State University,	US	5	153	2.35	80
Gupta, Kapil Kumar	U.P. Pandit Deen Dayal Upadhyaya pashu Chikitsa Vigyan Vishwavidyalaya Evam Go Anusandhan Sansthan	India	5	45	0	0
Sharma, Barkha D.	G.B. Pant University of Agriculture and Technology	India	5	33	0.06	1
Singh, Anubhav	U.P. Pandit Deen Dayal Upadhyaya pashu Chikitsa Vigyan Vishwavidyalaya Evam Go Anusandhan Sansthan	India	5	63	0	0
André, Marcos R.	Universidade Estadual Paulista Júlio de Mesquita Filho	Brazil	4	78	1.26	17
Baneth, Gad	Hebrew University of Jerusalem,	Israel	4	62	3.28	20
Chandrashekar, Ramaswamy	IDEXX Laboratories	US	4	79	2.67	45

**Figure-1:**
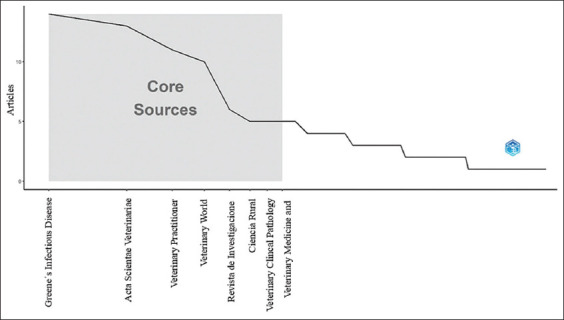
Core sources.

It was found that Zone 1 (core) included sources such as Greene’s Infectious Diseases of the Dog and Cat, Fifth Edition, and Acta Scientiae Veterinariae, which had the highest frequency of publications. Zone 2 included sources such as Veterinary Sciences and Animals. Finally, Zone 3 (periphery) included sources with a lower frequency of publications, such as Indian Veterinary Journal and Journal of Advanced Veterinary Research. This distribution is consistent with Bradford’s law, which suggests that some sources produce the most publications, whereas many sources produce only a few (Figure-1).

**Figure-2:**
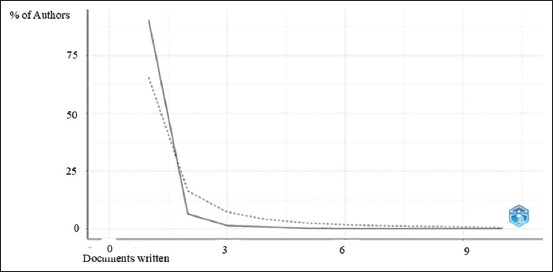
Author productivity.

The bibliometric study applied Lotka’s law to the authors’ production. According to the results, 983 authors published one paper, 70 authors published two papers, and so on. In this case, some authors produce the most publications, whereas most authors produce only a few publications (Figure-2).

**Figure-3:**
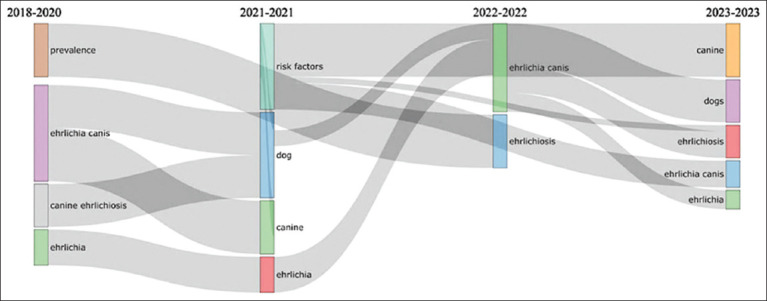
Thematic evolution.

The thematic evolution of the bibliometric study revealed several research focus changes. Between 2018 and 2020, the main topics were “canine ehrlichiosis” and “*Ehrlichia canis*” which appeared 3 and 5 times, respectively. These topics evolved to “dog” and “canine” in 2021, with 18 and 5 occurrences, respectively. In 2022, the focus shifted to “ehrlichiosis” with eight occurrences. By 2023, the themes of “canine,” “*Ehrlichia canis*” and “ehrlichiosis” continued to be relevant, but new themes such as “thrombocytopenia,” “dogs,” and “*Ehrlichia*” also emerged, indicating a change in research direction (Figure-3).

## Discussion

The genus *Ehrlichia* consists of five identified species: *E. canis*, *Ehrlichia chaffeensis*, *Ehrlichia ewingii*, *Ehrlichia muris*, and *Ehrlichia ruminantium*. At present, new *Ehrlichia* species are being discovered in different areas and types of ticks, expanding their distribution. Factors such as host population, migration, climatic changes, and control failures are environmental factors known to intensify the spread of *Ehrlichia* species [13]. The elevated serum globulin levels observed in this study could be attributed to a humoral response triggered by the continuous presence of *Ehrlichia* in the body [14]. This is supported at the high rate of seropositivity. As noted in the existing literature [15], such an antigenic stimulus, which results from the humoral response in a range of diseases (both infectious and non-infectious), is a well-known factor leading to glomerulopathy and subsequent proteinuria. This finding is consistent with our observations: Increased globulin levels, kidney damage, and proteinuria in the group of animals that were infected [16].

Blood testing remains an effective diagnostic method for clinical ehrlichiosis in dogs, and microscopic evaluation remains the simplest and most accessible diagnostic test in most laboratories [17]. The clinical diversity of ehrlichiosis and its presentation in different animal species is major topics of discussion. Although this disease has been extensively investigated in dogs and in less studied species such as cats, cattle, and horses, our understanding of the disease is still developing. This clinical variability presents challenges in diagnosis because symptoms can be generic and overlap with other common veterinary diseases [18]. The evolutionary processes leading to the development of the calmodulin-ubiquitin (CUB) gene cluster remain uncertain and are not the focus of this study. Nonetheless, the genetic variation of *E. canis trp36* has been linked to episodic selection events that are not uniformly distributed across nucleotide positions [19].

Aziz *et al*. [20] mentioned that *E. canis* does not show a preference for age or sex, but it has been observed that Siberian Huskies and German Shepherds tend to exhibit more severe symptoms. Effective treatments for canine ehrlichiosis include doxycycline, rifampicin, and minocycline. Their review succinctly described *Ehrlichia* infection in dogs and its prevalence in East and South Asian countries, discussed recent advances in chemotherapy and vectors responsible for disease transmission and highlighted areas requiring further research.

Similarly, Sainz *et al*. [21] emphasize that canine ehrlichiosis and anaplasmosis are significant tick-borne diseases with global distribution. There has been a recent increase in the amount of information and publications on these infections in Europe. The prevalence rates of *Ehrlichia* and *Anaplasma* spp. infections in dogs in several European countries are high. Mafruchati *et al*. [22] conducted their bibliometric study to analyze how the topic of diseases is addressed in veterinary textbooks, focusing on the chicken embryo, as well as the evolution of this topic. They collected metadata from 90 books downloaded from Scopus in CSV format. To analyze the data, they used the VOSviewer and biblioshiny tools of R Studio software, which enabled them to observe the evolution of the topic, citations, and number of book pages. In addition, the authors conducted a literature review to examine how the disease was represented in the samples. Their findings showed that the authors’ keywords, “heart” and “disease,” were strongly linked to the keyword “chicken embryo.” The keywords used in their study were “cells/cell,” “gene,” and “human.” These findings suggest that chicken embryo cells play an important role in determining their resistance to disease.

Colombino *et al*. [23] conducted a study to track the progression and identify potential gaps in veterinary intestinal health research over the past two decades (2000–2020). The researchers used the Web of Science database for their research and analyzed the results using the R package Bibliometrix. The study found a steady annual increase of 22.4% in the number of publications, with 1696 documents collected during the evaluation period. Most of the research focused on swine (34.8%), poultry (including chicken, duck, turkey, and quail – 33.9%), and aquaculture species (such as fish, crustaceans, and frogs – 15.0%). However, there have been fewer studies on felines, cows, horses, rodents, goats, and sheep. This comprehensive bibliometric analysis offers valuable insights into the development of this field and can serve as a guide for future research [23].

Finally, this study has certain limitations. First, the existing literature on Ehrlichiosis is scarce. Therefore, further research is necessary to deepen the analysis and corroborate the results obtained. The second limitation is that our study focused on exploring the correlation between ehrlichiosis. Therefore, further research on this highly relevant topic is essential. Despite these limitations, it is important to highlight that Scopus, with a larger number of journals and publications than other databases, ensures that most of the publications related to our study topic are represented [24, 25].

## Conclusion

This scientometric study provides a comprehensive overview of Ehrlichiosis research in veterinary medicine. First, the presence of international collaboration in 23% of the papers suggests that Ehrlichiosis is a global problem requiring a collaborative approach. This underscores the need to further encourage international collaboration in Ehrlichiosis research to share knowledge and resources. In addition, the thematic evolution from “canine ehrlichiosis” and “*Ehrlichia canis*” to “dog” and “canine” indicates a shift in research focus. This suggests that researchers are broadening their focus to include a broader range of animal species and clinical contexts. Veterinarians and researchers should consider this trend when developing prevention and treatment strategies. Finally, the concentration of productivity among some sources and authors, consistent with Bradford and Lotka’s laws, suggests that there are opportunities for more researchers and journals to contribute to the field. This approach could help diversify and enrich Ehrlichiosis research in veterinary medicine.

## Authors’ Contributions

FE, JM, FM, CM, RM, DG, and JP: Conception of the study. FM, JP, and FE: Extracted, verified, and analyzed the data and drafted and revised the manuscript. All authors have read, reviewed, criticized, and approved the final manuscript.
